# Differences in Glycemic Abnormalities Between Primary Aldosteronism and Essential Hypertension: A Systematic Review and Meta-Analysis

**DOI:** 10.3389/fendo.2022.870047

**Published:** 2022-03-31

**Authors:** Worapaka Manosroi, Pichitchai Atthakomol, Pittaporn Wattanawitawas, Supawan Buranapin

**Affiliations:** ^1^ Division of Endocrinology, Department of Internal Medicine, Faculty of Medicine, Chiang Mai University, Chiang Mai, Thailand; ^2^ Orthopaedics Department, Faculty of Medicine, Chiang Mai University, Muang Chiang Mai, Chiang Mai, Thailand; ^3^ Clinical Epidemiology and Clinical Statistic Center, Faculty of Medicine, Chiang Mai University, Chiang Mai, Thailand

**Keywords:** primary aldosteronism, essential hypertension, diabetes mellitus, impaired fasting glucose, impaired glucose tolerance, insulin resistance

## Abstract

**Background:**

The relationship of glycemic abnormalities between primary aldosteronism (PA) patients and essential hypertension (EH) patients is still debatable. This meta-analysis aimed to explore differences in the prevalence of glycemic abnormalities and levels of abnormal glucose metabolism in PA and EH patients.

**Methods:**

A search was performed using *PubMed*, *Scopus, Cochrane* and *Web of Science* databases from their inception through January 2022. Inclusion criteria for this study were 1) observational studies which contained specific data of interest, 2) studies including data which compared adult PA and EH patients and 3) studies which used appropriate methods to diagnose PA. Risk ratio (RR) or standardized mean difference (SMD) with a 95% confidence interval (95% CI) was calculated.

**Results:**

Twenty-six studies involving 53,186 patients were included in the meta-analysis. Patients with PA demonstrated significantly higher overall incidence of glycemic abnormalities than patients with EH [RR 1.54; 95% CI (1.20,1.98)]. Risk of diabetes mellitus (DM) and impaired glucose tolerance (IGT) in PA patients were higher than in EH patients [RR 1.27; 95%CI (1.08, 1.49) and RR 2.99; 95%CI (1.74, 5.16), respectively]. There was no statistically significant difference of risk between these groups for impaired fasting glucose (IFG) [RR 1.70; 95%CI (0.55, 5.26)]. Moderate heterogeneity was observed in overall glycemic abnormalities outcomes. A high level of heterogeneity was observed for IFG, while the level was low for DM and IGT.

**Conclusions:**

PA patients have a higher risk of glycemic abnormalities than in EH patients. Further study should be conducted to investigate underlying mechanisms of glycemic abnormalities in PA.

**Systematic Review Registration:**

www.inplasy.com, INPLASY, identifier 202220004.

## Introduction

Primary aldosteronism (PA) is the most common form of secondary hypertension. The reported prevalence in hypertensive patients varies from 7% to 33% depending on patient inclusion criteria, diagnostic methods and severity of hypertension (1–3). PA can be caused by either aldosterone producing adenoma (APA) or idiopathic hyperaldosteronism (IHA). PA is characterized by low renin and inappropriately high serum aldosterone levels which can lead to increased sodium resorption as well as potassium and hydrogen ion secretion. These mechanisms can result in resistant hypertension, hypokalemia and metabolic alkalosis which are the common presentations of PA (4). Apart from the effect of sodium retention and electrolyte dysregulations, studies have demonstrated that PA is also related to endothelial dysfunction, fibrosis, increased oxidative stress and increased risk of cardiac fibrosis (5, 6). These adverse mechanisms may explain the significantly increased risk of cardiovascular and cerebrovascular diseases in PA compared to essential hypertension (EH) patients (7, 8).

Another possible pathophysiology underpinning increased cardiovascular events in PA patients is abnormal glucose metabolism. A large body of evidence from *in vivo* studies has revealed that increased aldosterone levels can suppress insulin action and impair insulin responsiveness (9). Additionally, a study has demonstrated that insulin added *in vitro* to glomerulosa cells can produce an increase in aldosterone production (10). Increased prevalence of diabetes mellitus (DM), impaired glucose tolerance (IGT), and insulin resistance is more commonly observed in PA patients than in EH patients or in the normal population (11–13). Supporting these findings, a significantly decreased prevalence of DM and decreased fasting blood glucose (FBG) levels has been reported in PA patients following successful adrenalectomy (11). However, one study reported that the risk of impaired fasting glucose (IFG) was significantly lower in PA than in EH patients, while the prevalence of DM was not significantly different between the two groups (14). The upshot is that the relationship between glycemic abnormalities and PA has yet to be definitively determined.

A 2014 meta-analysis of 16 studies conducted by Chen et al. reported that abnormal glucose metabolism was more prevalent in PA than in EH patients (15). However, that study contained multiple methodological flaws (16). The present systematic review and meta-analysis aimed to systemically update findings regarding differences in the incidence of glycemic abnormalities in PA and EH patients. Other glucose metabolic parameters to assess insulin resistance and insulin secretion were also evaluated in this meta-analysis. This study also explored the sources of heterogeneity using subgroup and meta-regression analysis.

## Materials and Methods

### Search Strategy and Selection Criteria

This study followed the Preferred Reporting Items for Systematic Reviews and Meta-analyses (PRISMA) guidelines (17). The predefined protocol was registered in INPLASY 202220004. A comprehensive search of four databases, *PubMed/Medline*, *Scopus*, *Cochrane* and *Web of Science*, was performed from their inception to 4 January 2022. The keywords included were “hyperaldosteronism OR primary aldosteronism OR aldosteronism” AND “glucose OR insulin OR diabetes mellitus OR insulin resistance OR glucose metabolism OR glucose tolerance OR HOMA OR impaired glucose”. Medical subject heading (MeSH) terms were employed in the *PubMed*/*Medline* search. Details of the search strategy are provided in the **Supplementary Appendix**. Manual searches were performed by identifying references from the included studies, other relevant publications, and non-included reviews, and these were included as additional studies for the initial screening. Rayyan, a web-based program (Rayyan Systems Inc., Cambridge, MA, USA) (18), was employed for duplicate removal and initial screening of abstracts and titles.

Two authors (WM, PA) independently conducted the searches, screened for titles and abstracts. Pertinent studies were retrieved and underwent full-text screening for inclusion criteria. Then the two authors independently evaluated the methodological quality of the included studies and conducted the data extraction. The third author (PW) together with the first two authors (WM, PA) discussed and reached a consensus in cases of disagreement during the article search and selection processes.

Inclusion criteria for articles were as follows: 1) observational (non-randomized) and comparative studies of PA and EH patients age over 18 years; 2) studies containing at least one of the following: data on prevalence of abnormal glucose metabolism which were percentage of DM, IFG, IGT; levels of FBG, HbA1c; 2-hr oral glucose tolerance test (OGTT); homeostatic model assessment of insulin resistance (HOMA-IR); homeostatic model assessment of ß-cell function (HOMA-ß); area under the curve (AUC) of glucose; AUC of insulin and the quantitative insulin-sensitivity check index (QUICKI). These data should be compared between PA and EH patients and be presented as outcomes of interest, baseline characteristics or baseline investigations; and 3) use proper methods as recommended by standard guidelines to diagnose and confirm PA (19). Exclusion criteria were articles published in a language other than English, review articles, case reports, grey literature, editorial comments, conference abstracts and animal studies. Studies involving special populations such as pregnant women or children were also excluded. In cases of multiple publications involving the same cohort, only the one which contained the highest number of patients was chosen.

### Data Extraction

Data extraction was independently conducted by two authors (WM, PA) using predesigned Microsoft Excel spreadsheets. The demographic variables extracted from each study were: 1) study characteristics, i.e., the name of the first author, year of publication, ethnicity of the included population, study design, whether the study was demographically matched between case (PA) and control (EH), the number of PA (case) and EH (control) patients and the criteria of screening and confirmation for PA diagnosis; 2) patient characteristics and potential confounders, i.e., means and standard deviations (SD) of age, percentage of the predominant sex, predominant ethnicity and mean BMI; 3) outcomes of interests including percentage of abnormal glucose metabolism including DM, IFG and IGT; glucose metabolic parameters including mean and SD of FBG, HbA1c, 2-hr OGTT, HOMA-IR, HOMA-ß, AUC of glucose, AUC of insulin and QUICKI for each group of PA and EH patients. For studies which did not provide data on HOMA-IR, fasting glucose and fasting insulin levels, if available, were used to calculate this value. All the mean values were converted to and are reported as SI units.

### Data Synthesis

The meta-analysis was performed using the STATA program version 15.0. (StataCorp, College Station, TX, USA). Comparison between PA and EH, risk ratio (RR) with 95% confidence interval (95% CI), was calculated to assess the effect size for outcomes which were binomial proportions (percentage of events). Standardized mean difference (SMD) with a 95% CI was calculated for continuous outcome variables (glucose metabolic parameters). Additionally, the proportion of patients with any abnormal glucose metabolism in each study was combined to calculate a pooled prevalence of all glycemic abnormalities from all studies. For studies which provided data on more than one abnormal glucose metabolism, only the prevalence of DM or IFG (if prevalence of DM was not provided) or IGT (if the prevalence of DM and IFG were not provided) was chosen for the pooled analysis of all glycemic abnormalities. For FBG outcome, only the studies which excluded DM patients or instructed the patients to discontinue hypoglycemic agents before FBG measurement were included for analysis. Random-effect modelling by DerSimonian-Liard method was performed in this study as the observed estimates of treatment effects can vary across studies due to real differences in the treatment effects in each research and sampling variability. The statistical significance level for this meta-analysis was set at p<0.05. To evaluate the statistical heterogeneity among the studies, the I^2^ statistic was assessed. I^2^ values of >75% with a significant Cochran Q test (p<0.05) were considered to indicate high heterogeneity. I^2^ values of < 25-50% and >50-75% were considered as low and moderate heterogeneity, respectively. Publication bias was assessed using funnel plots and Egger’s linear regression tests. A p-value of <0.05 was considered to indicate statistically significant of publication bias for Egger’s regressions. The asymmetry of funnel plot also indicated publication bias.

In the pooled prevalence of all forms of abnormal glucose metabolism, further analysis was also conducted to determine the effect of the potential confounders which were sex, age, ethnicity, BMI, and demographic data matching. Categorical variables including ethnicity and demographic data analysis were analyzed by subgroup analyses. Continuous variables including age, sex, and BMI were analyzed by meta-regression analyses. Age and sex were further analyzed because some studies reported differences in glucose metabolic profiles between genders and among different age groups (20). For ethnicity, comparison was made between Asians and non-Asians as a study has reported a difference in the prevalence of glycemic abnormalities and glucose metabolic parameters between Asians and non-Asians (21). Regarding BMI, further analysis was performed as a study has shown that blood glucose is positively correlated with BMI (22). To control for bias from confounders, subgroup analysis was also performed by categorizing studies into those with and without demographic data matching. Studies with at least one matched variable (sex, age, BMI, blood pressure or duration of hypertension) were categorized into subgroups with demographic data matching.

### Risk of Bias Assessment

Joanna Briggs Institute (JBI) Critical Appraisal Tools for cohort, case-control and cross-sectional assessment of the risk of bias was conducted by two independent authors (WM and PA) and discrepancies were resolved through discussion with the third author (PW) The details of JBI criteria checklists are available elsewhere (23).

### Certainty of the Evidence

The quality of the evidence provided was graded independently by two authors (WM and PA) using the Grading of Recommendation, Assessment Development and Evaluation (GRADE) tool (24). In brief, grading of evidence began with assessment of the quality of the study design which was low for observational studies and high for randomized controlled trials. Then the certainty of the evidence was considered to be increased or decreased based on multiple conditions, e.g. study limitations, consistency of effect, imprecision, indirectness, publication bias, magnitude of the effect, dose-response gradient, and whether any of the plausible confounders would alter the effect. The level of certainty of evidence was categorized as “high”, “moderate”, “low” or “very low”. Any disagreements were resolved by the third author (PW).

## Results

**Figure 1:**
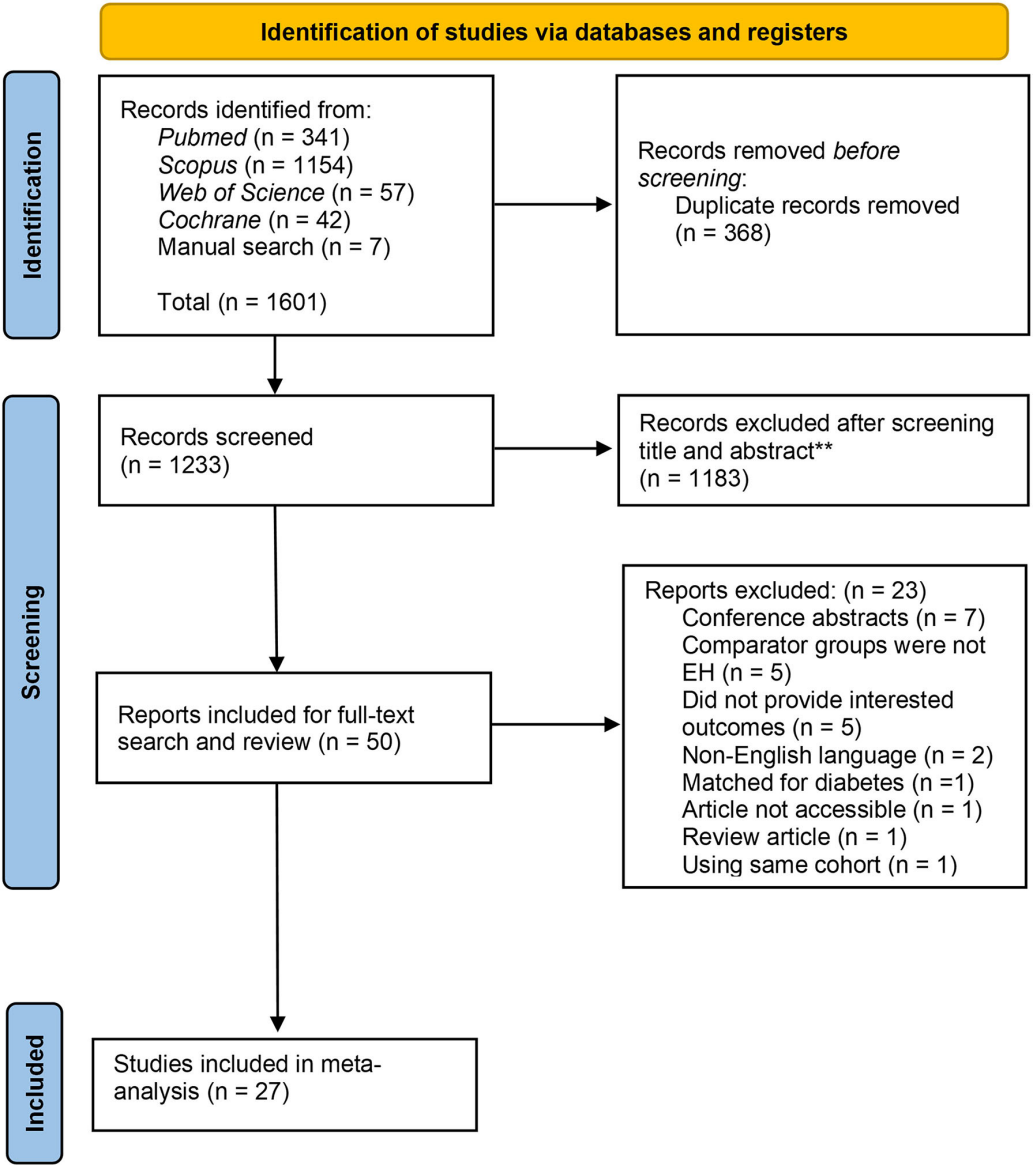
PRISMA flow diagram.

A total of 1601 articles were retrieved from database searches including 341 from *PubMed*, 1,154 from *Scopus*, 57 from *Web of Science*, 42 from *Cochrane* and 7 from manual searches. From the retrieved articles, 368 duplicates were removed. A screening of titles and abstracts of 1,233 articles were performed which resulted in the exclusion of 1,183 additional articles which were not relevant to the objectives of this study. The full texts of the remaining 50 articles were retrieved and reviewed resulting in the exclusion of an additional 23 articles due to multiple reasons including conference abstracts, comparator groups not EH, already matched for diabetes, did not provide outcomes of interest, written in a language other than English, full text not accessible, and review articles using the same cohort as an included study. Finally, a total of 27 studies were included (2, 13, 14, 25–48). The study selection process is shown in **Figure 1**.

### Study Characteristics

**Table 1** shows the characteristics of the included studies. The 27 studies included in this meta-analysis demonstrate differences in prevalence of glycemic abnormalities or glucose metabolic parameters in PA and EH patients. All the studies were non-randomized, and most were cross-sectional studies. The majority of the studies were conducted in non-Asian populations. All studies provided data on age, ethnicity, and BMI. One study did not provide data on sex (47). Nineteen studies were matched for demographic data (13, 14, 26, 27, 30–36, 38, 39, 41–45, 47). Regarding outcomes, 15 studies provided data on the prevalence of DM (2, 13, 14, 27–29, 31, 32, 34, 35, 37–39, 47, 48), 4 studies on IFG (2, 14, 35, 40) and 5 studies on IGT (25, 28, 35, 36, 47). Twenty studies included data on FBG (2, 14, 25, 27, 28, 30, 32, 34–38, 40–47) with 10 studies excluded DM patients or discontinuing hypoglycemic agents before recruitment (25, 30, 36, 40–46), 7 on HbA1c (13, 25, 26, 30, 31, 36), 5 on 2-hr OGTT (25, 30, 32, 36, 47), 10 on HOMA-IR (25, 26, 28, 30, 33, 36, 41–44), 5 on HOMA-ß (26, 30, 33, 43, 45), 3 on AUC of glucose (25, 26, 45), 4 on AUC of insulin (25, 26, 36, 45) and 3 on QUICKI (25, 33). The criteria for screening and confirmation tests for PA diagnosis are provided in the **Supplementary Appendix**.

**Table 1:** Baseline characteristics of the included studies.

Author	Year	Type of studies	Number of patients (PA/EH)	Male (%)	Mean age ± SD (years)	Mean BMI ± SD (kg/m2)	Ethnic	Demographic data matching	Interested outcomes	Definition of glycemic abnormalities	JBI risk of bias
**Grewal et al.** (25)	2021	Cross-sectional	21/22	41.9	52.8 ± 17.7	31.2 ± 1.9	Non-Asian	No	IGT, FBG, HbA1c, HOMA-IR, AUC glucose, AUC insulin, 2hr OGTT, QUICKI, Matsuda index	N/A	Moderate
**Huang et al.** (26)	2021	Retrospective	174/174	48.3	46.2 ± 12.6	24.7 ± 3.9	Asian	Yes	HbA1c, HOMA-IR, HOMA-ß, AUC glucose, AUC insulin	N/A	Low
**Choudhary et al.** (27)	2020	Cross-sectional	130/130	64.6	52.9 ± 16.9	29.9 ± 9.7	Non-Asian	Yes	DM, FBG	N/A	Low
**Manosroi et al.** (28)	2020	Cross-sectional	41/38	35.4	38.4 ± 14.5	29.6 ± 6.8	Asian	No	DM, FBG, HOMA-IR	N/A	Low
**Vujacik et al.** (29)	2020	Retrospective	40/40	35	52.8 ± 11.8	28.1 ± 4.5	Non-Asian	No	DM, IGT	N/A	Moderate
**Zhang et al.** (30)	2020	Cross-sectional	109/109	40.4	45 ± 9.6	24.1 ± 3.8	Asian	Yes	FBG, HbA1c, HOMA-IR, HOMA-ß, 2hr OGTT	N/A	Low
**Hundemer et al.** (31)	2018	Cross-sectional	602/41853	48.7	57 ± 12	29.8 ± 6.4	Non-Asian	Yes	DM, HbA1c	N/A	Low
**Monticone et al.** (2)	2017	Cross-sectional	99/1573	56.5	46.1 ± 8.9	26.1 ± 4.3	Non-Asian	No	DM, IFG, FBG	N/A	Low
**Murata et al.** (48)	2017	Cross-sectional	292/498	51.9	60.9 ± 12.2	24.1 ± 3.4	Asian	No	DM	N/A	Low
**Yang et al.** (32)	2016	Cross-sectional	100/100	58	50 ± 12	25.4 ± 3.3	Asian	Yes	DM, FBG	N/A	Low
**Watanabe et al.** (33)	2016	Cross-sectional	32/21	35.8	56 ± 12.3	23.5 ± 4.5	Asian	Yes	HOMA-IR, HOMA-ß, QUICKI	N/A	Low
**Hanslik et al.** (13)	2015	Prospective	250/250	64.4	52.6 ± 11.1	28.6 ± 4.9	Non-Asian	Yes	DM, HbA1c	N/A	Low
**Turchi et al.** (34)	2014	Prospective	66/132	45.4	52.1 ± 10.5	27.2 ± 3.6	Non-Asian	Yes	DM, FBG	N/A	Moderate
**Savard et al.** (35)	2013	Retrospective	459/1290	65.7	51.2 ± 10.3	27.9 ± 5.0	Non-Asian	Yes	DM, IFG, IGT, FBG	N/A	Low
**Fischer et al.** (36)	2013	Cross-sectional	23/11	60.6	52 ± 13.3	29.3 ± 4.3	Non-Asian	Yes	IGT, FBG, HOMA-IR	IFG: FBG >5.6 mmol/L	Low
**Prejbisz et al.** (37)	2013	Cross-sectional	32/172	60.3	48.4 ± 7.7	30.1 ± 4.7	Non-Asian	No	DM, FBG	N/A	Moderate
**Somloova et al.** (38)	2010	Retrospective	100/90	54.2	49.9 ± 10.1	28.7 ± 4.6	Non-Asian	Yes	DM, FBG	N/A	Low
**Reinke et al.** (39)	2010	Prospective	338/338	59.4	50 ± 0	28 ± 0	Non-Asian	Yes	DM	N/A	Low
**Iacobellis et al.** (40)	2010	Cross-sectional	75/192	55.0	54.9 ± 12.4	26.9 ± 3.7	Non-Asian	No	IFG, FBG	IFG: FBG >5.6 mmol/L	Low
**Ronconi et al.** (41)	2009	Cross-sectional	89/164	52.1	51 ± 11	27.4 ± 4.2	Non-Asian	Yes	FBG, HOMA-IR	N/A	Low
**Fallo et al.** (42)	2010	Cross-sectional	40/40	65	52 ± 9	26.7 ± 2.8	Non-Asian	Yes	FBG, HOMA-IR	N/A	Low
**Matrozova et al.** (14)	2009	Retrospective	460/1363	66.2	51.7 ± 10.4	27.3 ± 4.6	Non-Asian	Yes	DM, IFG, FBG	IFG: FBG 5.6-7 mmol/L IGT: OGTT 7.8-11.1 mmol/L	Low
**Mosso et al.** (43)	2007	Cross-sectional	30/60	27.8	57.8 ± 9.5	29.8 ± 5.3	Non-Asian	Yes	FBG, HOMA-IR, HOMA-ß	N/A	Low
**Fallo et al.** (44)	2007	Cross-sectional	40/40	60	52 ± 10.5	27.1 ± 3.9	Non-Asian	Yes	FBG, HOMA-IR	N/A	Low
**Catena et al.** (45)	2006	Prospective	47/274	70.4	53 ± 12	28.4 ± 2.8	Non-Asian	Yes	FBG, HOMA-ß, AUC glucose, AUC insulin, QUICKI	N/A	Moderate
**Fallo et al.** (46)	2006	Prospective	85/381	57.2	53.4 ± 12.4	27.8 ± 4.5	Non-Asian	No	FBG	N/A	Low
**Widimsky et al.** (47)	2001	Cross-sectional	36/21	N/A	52.4 ± 9.2	28.1 ± 4.3	Non-Asian	Yes	DM, IGT, 2hr OGTT	IGT: OGTT 7.8-11.1 mmol/L DM: OGTT ≥11.1 mmol/L	Low

PA, Primary aldosteronism; EH, Essential hypertension; SD, Standard deviation; N/A, Not available; DM, Diabetes mellitus; IFG, Impaired fasting glucose; IGT, Impaired glucose tolerance; FBG, Fasting blood glucose; HOMA-IR, Homeostatic model assessment of insulin resistance; HOMA-ß, Homeostatic model assessment of beta cell function; AUC, Area under the curve; OGTT, oral glucose tolerance test.

### Risk of Bias in the Studies

Risk of bias was assessed using JBI tools for cohort, case-control and cross-sectional studies (**Table 1** **)**. Most of the studies (22 of 27) evidenced high quality with a low risk of bias (2, 13, 14, 26–28, 30–33, 35, 36, 38–44, 46–48). The other five studies (25, 29, 34, 37, 45) showed moderate quality with a moderate risk of bias due to 1) important confounders not being identified and appropriately controlled for by proper statistical analyses for cross-sectional studies and 2) not being evaluated regarding the outcomes issues as measured outcomes were not relevant to the primary objectives of the current study for cohort studies. Details of the risk of bias scores for each study are shown in the **Supplementary Appendix**.

### Results of Syntheses

**Figure 2:**
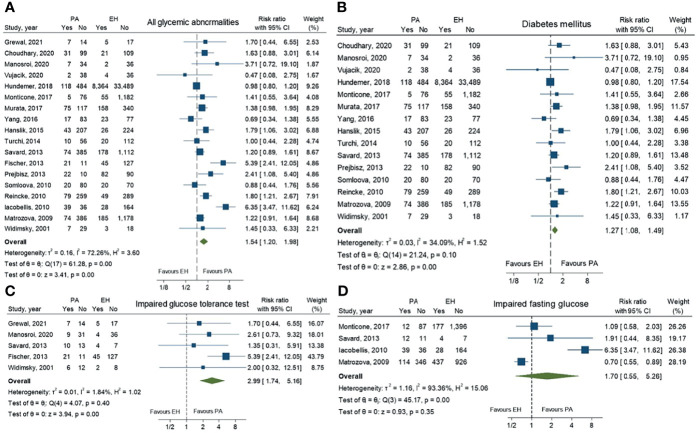
Forest plots comparing risk ratio of all glycemic abnormalities **(A)**, diabetes mellitus **(B)**, impaired glucose tolerance **(C)** and impaired fasting glucose **(D)** between primary aldosteronism and essential hypertension patients.

A total of 27 studies comprising 53,186 patients were included in this meta-analysis. In the analysis of pooled prevalence of all glycemic abnormalities, patients with PA demonstrated a significantly higher risk than patients with EH [RR 1.54; 95% CI (1.20,1.98)]. Analyzed separately, PA patients had a significantly increased risk of DM and IGT compared to EH patients [RR 1.27; 95%CI (1.08, 1.49) and RR 2.99; 95%CI (1.74,5.16), respectively]. For IFG, there was no statistically significant difference of risk between PA and EH patients [RR 1.70; 95%CI (0.55,5.26)]. Data are shown in **Figure 2**.

**Figure 3:**
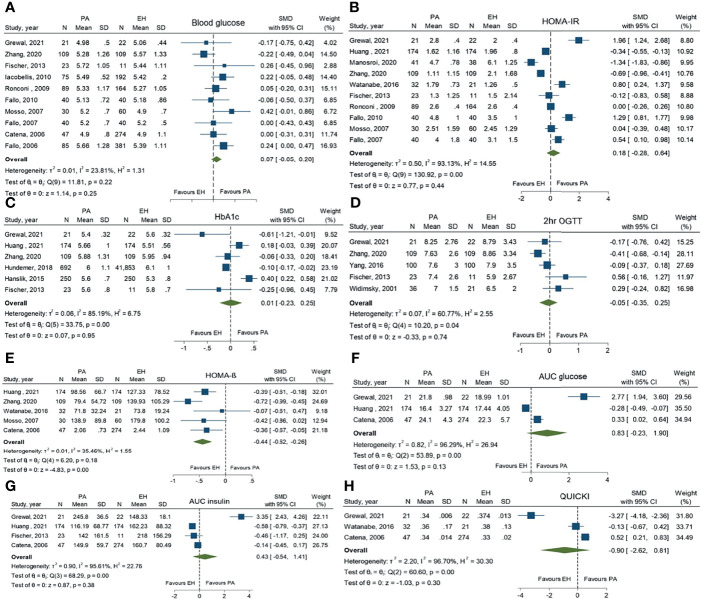
Forest plots comparing glucose metabolic profiles of primary aldosteronism and essential hypertension patients: fasting blood glucose **(A)**, homeostatic model assessment of insulin resistance (HOMA-IR) **(B)**, HbA1c **(C)**, 2-hr oral glucose tolerance test (2-hr OGTT) **(D)**, homeostatic model assessment of ß-cell function (HOMA-ß) **(E)**, area under the curve (AUC) of glucose **(F)**, AUC of insulin (G) and the quantitative insulin-sensitivity check index (QUICKI) **(H)**.

For glucose metabolic parameters, PA patients had significantly lower levels of HOMA-ß than EH patients (SMD -0.44; 95%CI (-0.62, -0.26)). Other parameters, including FBG, HbA1c, 2-hr OGTT, HOMA-IR, AUC of glucose, AUC of insulin and QUICKI, revealed no statistically significant difference between the two groups (**Figure 3** **).**

### Subgroup Analysis and Meta-Regression Analysis

Subgroup analysis was performed on the pooled prevalence of all glycemic abnormalities. In the analysis of ethnicity, only non-Asian subgroups showed a prevalence of glycemic abnormalities that was significantly higher in PA than in EH patients (RR 1.62; 95%CI (1.22,2.15). Regarding demographic data matching, in both matched and unmatched subgroups PA patients had higher risk of glycemic abnormalities than EH patients [RR 1.33; 95%CI (1.04,1.69) and RR 2.08; 95%CI (1.11, 3.88), respectively]. Data are as shown in **Table 2** and the **Supplementary Appendix**. Both univariate and multivariate meta-regression showed that the association of all glycemic abnormalities between PA and EH was not varied by age, sex, or BMI. Data are as shown in **Table 3**.

**Table 2:** Subgroup analysis for assessment of all glycemic abnormalities between primary aldosteronism and essential hypertension patients.

**Subgroup**	**Number of analyses**	**RR (95%CI)**	**I^2^ (%)**	**I^2^ p-value**
Ethnicity				
- Asian-predominant	3	1.22 (0.63, 2.36)	58.6	0.09
- Non-Asian- predominant	15	1.62 (1.22, 2.15)	75.1	<0.001
Demographic data matching				
- Unmatched	7	2.08 (1.11, 3.88)	73.1	<0.001
- Matched	11	1.33 (1.04, 1.69)	64.4	<0.001

**Table 3:** Meta-regression analysis for assessment of all glycemic abnormalities between primary aldosteronism and essential hypertension patients.

Covariates	Univariate			Multivariate		
	Coefficient	95%CI	p-value	Coefficient	95%CI	p-value
Age	-0.01	(-0.09, 0.06)	0.75	-0.004	(-0.09, 0.08)	0.91
Sex	0.009	(-0.03, 0.050)	0.60	0.01	(-0.03, 0.05)	0.60
BMI	0.06	(-0.12, 0.24)	0.49	0.06	(-0.14, 0.27)	0.53

### Reporting of Heterogenicity

For pooled prevalence of all glycemic abnormalities, moderate heterogeneity among the studies was observed with an I^2^ of 72.3%. High heterogeneity among the studies was observed in IFG outcomes with an I^2^ of 93.4%. For DM and IGT outcomes, there was low heterogeneity among the studies with I^2^ values of 34.1% and 1.8%, respectively (**Figure 2**). For glucose metabolic parameter outcomes, HbA1c, HOMA-IR, AUC of glucose, AUC of insulin and QUICKI index showed high heterogeneity with I^2^ values of 85.2%, 93.1%, 96.3%, 95.6% and 96.7%, respectively. FBG and HOMA-ß showed low heterogeneity (I^2^ = 23.8% and 35.5%, respectively), while 2-hr OGTT showed moderate heterogeneity (I^2^ = 60.7%).

Subgroup analysis revealed that only ethnicity was the source of heterogeneity. Asian-predominant studies were more homogeneous than non-Asian predominant studies (I^2^ = 58.6% versus 75.1%). Analysis results are shown in **Table 2**. Meta-regression analysis to explore the source of heterogeneity among studies indicated that age, sex, and BMI might not be the origin of heterogeneity among studies as all p-values were >0.05. Data are as shown in **Table 3**.

### Publication Bias

Egger’s regression test did not reveal any publication bias for overall prevalence of glycemic abnormalities, prevalence of DM, IFG, IGT, differences of FBG, HbA1c, 2-hr OGTT, HOMA-IR, HOMA-ß, AUC of glucose, AUC of insulin or QUICKI. Similarly, the funnel plots for all outcomes mentioned were symmetrical. **(** **Supplementary Appendix** **).**

### Certainty of the Evidence

According to the GRADE assessment for certainty of the evidence, all the outcomes showed very low certainty of the evidence because all the outcomes were achieved from observational studies which were initially rated as having a low quality of evidence. All the outcomes were considered indirect (downgrade one level) and most of the outcomes were considered inconsistent due to large heterogeneity (downgrade one level) and imprecise due to the wide CI with threshold crossing (downgrade one level). Other aspects required no rating change. Thus, the overall level of certainty was very low **(** **Supplementary Appendix** **).**

## Discussion

This systematic review and meta-analysis revealed results of prevalence of glycemic abnormalities and differences in glucose metabolic profiles between PA and EH patients. Based on the pooled prevalence of glycemic abnormalities, the present study found the prevalence of DM and IGT were higher in PA than in EH patients, while the prevalence of IFG showed no significant difference. The study also found significantly lower HOMA-ß levels in PA than EH patients, while other glucose metabolic parameters did not show any significant differences between the groups.

An enhanced risk of atherosclerotic cardiovascular and cerebrovascular diseases in PA patients has been reported (7, 8, 49). A theory has been proposed that in PA, excess aldosterone can lead to arterial wall thickening and fibrosis, and that increased oxidative stress and inflammation can result in endothelial dysfunction (50). Additionally, a study in the German Conn’s registry demonstrated that DM is more commonly observed in PA patients than in a matched normal population which could be a partial explanation of the increased cardiovascular risk in PA patients (13). Increased glucose levels in DM patients can cause atherosclerotic cardiovascular disease through multiple mechanisms including polyol, advance glycation end products, protein kinase C and hexosamine pathways (51).

Two previous meta-analyses comparing the incidence of glycemic abnormalities in PA and EH have been conducted. Chen et al. in 2013 performed a meta-analysis which included 16 studies that found the prevalence of glycemic abnormalities in PA was higher than in EH patients with an OR of 1.55 and 95% CI = 1.01, 2.36 (15). Similarly, in our meta-analysis, PA patients had a 1.54-fold higher risk of having some form of glycemic abnormality than EH patients. In 2019, Wu et al. reported no significant difference in blood glucose level between PA and EH patients, a result similar to our meta-analysis (49). In their meta-analysis, only blood glucose outcome was reported with 9 studies included, while in our study, 1 more study was included for FBG outcome. Unlike our study, their study did not exclude studies that included DM patients, or those that instructed them to discontinue hypoglycemic agents before the recruitment period as these factors may affect the glucose measurement. Moreover, data on the differences in the prevalence of DM, IGT and IFG were not reported in their meta-analysis. In the Chen et al. study, the insulin resistance index for PA patients was higher than in the normal population but lower than in EH patients (15). In that study, the insulin sensitivity index was compared only to normal population, and it was lower in PA than in normal population. However, the meta-analysis from Chen et al. contained multiple methodological limitations (15, 16). First, Chen did not incorporate several relevant studies in the meta-analysis which could have affected the study outcomes. Second, the prevalence outcomes were pooled as a proportion rather than by using the quantitative data, leading to uninterpretable results. Third, the pooled prevalence of IFG had different definitions in different studies (e.g., fasting blood glucose over 5.6 in some studies and 6.1 mmol/L in others), which can cause statistical heterogeneity. Fourth, for the overall pooled results of glycemic abnormalities, multiple studies contributed more than once to the overall pooled result of glycemic abnormalities, e.g., both DM and IGT or IFG which led to the redundant results. These limitations suggest the Chen study results should be interpreted cautiously. The present meta-analysis has addressed all the aforementioned limitations.

Presently, the nature of the relationship between plasma aldosterone and glucose metabolism is still controversial. Multiple plausible aspects of physiology could potentially explain the increased risk of glycemic abnormalities in PA patients. First, excess aldosterone and mineralocorticoid activation can interfere with the multiple steps of the insulin signaling pathway, e.g., mitogen-activated protein kinase (MAPK) signaling, protein kinase B (Akt2) and the serum/glucocorticoid regulated kinase 1 (SGK1) pathway (9). Second, excess aldosterone can increase hepatic glucose production *via* increased glucose-6-phosphatase (G6Pase) and fructose-1,6-bisphosphatase and phosphoenolpyruvate carboxykinase (52). Third, based on an animal study, increased aldosterone levels can impair insulin secretion by directly affecting islet cell function (53). Fourth, aldosterone induces the expression of monocyte chemoattractant protein-1 (MCP-1), which is related to insulin resistance (54). Fifth, the concomitant presence of cortisol co-secretion with PA was demonstrated to be common than previously understood. The reported prevalence of this co-secretion ranged from 4 to 16% in PA (55). This may contribute to the higher prevalence of glycemic abnormalities in PA than in EH. However, the prevalence of co-secreting adenoma was not documented in all of the included studies. The final proposed mechanism is excess aldosterone levels can cause hypokalemia. Hypokalemia by itself can lessen insulin secretion as well as lead to insulin resistance though ATP-sensitive potassium channels on islet cells (56).

From the present meta-analysis, only the prevalence of DM and IGT has been demonstrated to be significantly higher in PA patients compared to EH patients. IFG has shown a comparable risk in both PA and EH patients. This could be explained by the statistical heterogeneity in the included studies as there was a wide variety of IFG definitions in those studies. HOMA-ß, a marker of ß-cell function, was the only glucose metabolic profile which was significantly higher in PA than in EH patients. Other markers of insulin resistance or insulin sensitivity such as HOMA-IR, QUICKI, AUC glucose and insulin were not significantly different between the two groups. However, according to the above-mentioned evidence of the pathophysiology of aldosterone and the insulin effect, both insulin resistance, sensitivity and ß-cell function are likely to be affected by excess aldosterone levels. We presumed that the non-significant relationships of other markers apart from HOMA-ß were the result of the small number of included studies and the wide heterogeneity among those studies.

Because of the moderate heterogeneity among the studies, results related to the main outcome of overall glycemic abnormalities in this meta-analysis should be interpreted cautiously. Regarding the heterogeneity issue, confounders which could potentially interfere with the glycemic abnormalities were addressed by subgroup and meta-regression analyses. The results of the subgroup analyses revealed that the risk of glycemic abnormalities was significantly higher in PA than in EH patients in studies which included a preponderance of non-Asians. A plausible explanation of the different outcomes in Asians and non-Asians is that non-Asian populations have a higher baseline of insulin resistance than Asians. When PA develops in these non-Asian population, the increase in aldosterone levels may result in worsening of insulin resistance and thus to a greater prevalence of glycemic abnormalities (57). However, this explanation is currently just a theory as the data on the association of ethnicity with glycemic abnormalities and plasma aldosterone has not yet been clearly elucidated.

A strength of this meta-analysis in addressing differences in glycemic abnormalities between PA and EH patients is that only studies which had clear criteria for the diagnosis of PA were included. Additionally, this meta-analysis addressed the limitation issues in previous meta-analyses as described above (15). Another strength is that subgroup and meta-regression analysis were performed to identify the effects of possible confounders and to recognize the causes of heterogeneity.

Several limitations in this study need to be acknowledged. First, there was a high level of heterogeneity among the included studies, especially as regards the main outcome. Second, as the primary objective of most of the studies did not include finding differences in glycemic abnormalities between PA and EH patients, most of the outcome data used in this meta-analysis were acquired from baseline characteristics. Because of that, some potential confounders such as sex, age and BMI were not matched prior to the analysis and may have affected the glycemic outcomes. However, the results of subgroup analysis by demographic data matching did show a higher incidence of glycemic abnormalities in both of the subgroups. Additionally, lifestyle, activity level, diet and genetics may possibly affect glycemic abnormalities, but most of the studies did not provide data concerning these factors.

## Conclusions

Compared to EH patients, PA patients have a significantly higher risk of glucose abnormalities. This indicates that one of the factors which lead to increased atherosclerotic cardiovascular events in PA patients may result from an increased prevalence of glycemic abnormalities. Future research is required to address the mechanisms and relationships of glucose metabolism and PA.

## Data Availability Statement

The raw data supporting the conclusions of this article will be made available by the authors, without undue reservation.

## Author Contributions

WM designed the study, collected, analyzed, and interpreted the data, and was the major contributor in writing the manuscript. PA collected and performed data analysis. PW and SB reviewed and edited the manuscript. All authors contributed to the article and approved the submitted version.

## Conflict of Interest

The authors declare that the research was conducted in the absence of any commercial or financial relationships that could be construed as a potential conflict of interest.

## Publisher’s Note

All claims expressed in this article are solely those of the authors and do not necessarily represent those of their affiliated organizations, or those of the publisher, the editors and the reviewers. Any product that may be evaluated in this article, or claim that may be made by its manufacturer, is not guaranteed or endorsed by the publisher.
