# Supplementary material for: Differences in Glycemic Abnormalities Between Primary Aldosteronism and Essential Hypertension: A Systematic Review and Meta-Analysis
Source: Front Endocrinol (Lausanne). 2022 Mar 31;13:870047. doi: 10.3389/fendo.2022.870047 (PMC9009178; doi:10.3389/fendo.2022.870047)

**Supplementary Appendix**

**Table S1.** Keywords for article searches

**Table S2.** Screening and confirmatory criteria of primary aldosteronism of the 27 include studies

**Table S3.1.** Risk of bias assessed by Joanna Briggs Institute (JBI) Critical Appraisal Tools for cross-sectional study

**Table S3.2.** Risk of bias assessed by Joanna Briggs Institute (JBI) Critical Appraisal Tools for cohort study

**Table S3.3.** Risk of bias assessed by Joanna Briggs Institute (JBI) Critical Appraisal Tools for case-control study

**Table S3.** Egger’s regression analysis

**Table S5.** Grading of Recommendations Assessment, Development and Evaluation (GRADE) rating

**Figure S1.** Forest plots of subgroup analysis of all glycemic abnormalities comparing primary aldosteronism and essential hypertension patients

**Figure S2.** Funnel plots of studies with outcomes of glycemic abnormalities

**Figure S3.** Funnel plots of studies with outcomes of glucose metabolic profiles

**Table S1.** Keywords for article searches

| **Keywords** | **Number of articles** |
| --- | --- |
| ***Pubmed*** | 341 |
| ("Hyperaldosteronism"[Mesh] OR "primary aldosteronism" OR "aldosteronism") AND ("Glucose"[Mesh] OR "Insulin"[Mesh] OR "Diabetes Mellitus"[Mesh] OR "insulin resistance" OR "glucose metabolism" OR "glucose tolerance" OR "HOMA" OR “impaired glucose”) |  |
| ***Scopus*** | 1,154 |
| (( "Hyperaldosteronism"  OR  "primary aldosteronism"  OR  "aldosteronism")  AND  ("Glucose"  OR  "Insulin"  OR  "Diabetes Mellitus"  OR  "insulin resistance"  OR  "glucose metabolism"  OR  "glucose tolerance"  OR  "HOMA"  OR  "impaired glucose")) |  |
| ***Web of science*** | 57 |
| (( "Hyperaldosteronism"  OR  "primary aldosteronism"  OR  "aldosteronism")  AND  ("Glucose"  OR  "Insulin"  OR  "Diabetes Mellitus"  OR  "insulin resistance"  OR  "glucose metabolism"  OR  "glucose tolerance"  OR  "HOMA"  OR  "impaired glucose")) |  |
| ***Cochrane*** | 42 |
| (("Hyperaldosteronism"  OR  "primary aldosteronism"  OR  "aldosteronism")  AND  ("Glucose"  OR  "Insulin"  OR  "Diabetes Mellitus"  OR  "insulin resistance"  OR  "glucose metabolism"  OR  "glucose tolerance"  OR  "HOMA"  OR  "impaired glucose")) |  |

**Table S2.** Screening and confirmatory criteria of primary aldosteronism of the 27 include studies

| **Author** | **Year** | **Screening criteria** | **Confirmatory criteria** |
| --- | --- | --- | --- |
| **Grewal** | 2021 | ARR >20-40 (ng/dL)/(ng/mL/hr)* | SIT  - post-infusion PAC >10 ng/dL  - post-infusion PAC (seated) >6 ng/dL |
| **Huang** | 2021 | - ARR >20-40 (ng/dL)/(ng/mL/hr)*  - 2016 China’s  primary aldosteronism treatment expert consensus (data not available) | SIT  - post-infusion PAC >10 ng/dL  - post-infusion PAC (seated) >6 ng/dL  CCT  - PAC suppressed <30% after captopril |
| **Choudhary** | 2020 | -PAC (pmol/L) to PRA (ng/mL/h) ratio >750, with PAC≥ 280 pmol/L  -PAC (pmol/L) to DRC (mU/L) ratio >30, with PAC ≥280 pmol/L | Oral sodium loading test  - Urine aldosterone excretion >33 nmol/day |
| **Manosroi** | 2020 | ARR >20 (ng/dL)/(ng/mL/hr) with PAC >15 ng/dL and suppressed PRA | SIT  - post-infusion PAC >10 ng/dL |
| **Vujacik** | 2020 | ARR >20-40 (ng/dL)/(ng/mL/hr)* | SIT  - post-infusion PAC >10 ng/dL  - post-infusion PAC (seated) >6 ng/dL  CCT  - PAC suppressed <30% after captopril |
| **Zhang** | 2020 | ARR >25 (ng/dL)/(ng/mL/hr) | CCT  - PAC >10 ng/dL with suppressed PRA or ARR>  8.1 (ng/dL)/(ng/mL/hr)  SIT  - post-infusion PAC >10 ng/dL |
| **Hundemer** | 2018 | ARR >20 (ng/dL)/(ng/mL/hr) | Oral sodium loading  - 24h urine aldosterone excretion > 33 nmol with 24h urine sodium excretion > 200 mmol  SIT  - Post-infusion PAC > 280 nmol/L |
| **Monticone** | 2017 | ARR >30 (ng/dL)/(ng/mL/hr) with PAC>10 ng/dL | SIT  - post-infusion PAC >5 ng/dL  CCT  - ARR >30 (ng/dL)/(ng/mL/hr) |
| **Murata** | 2017 | ARR >20 (ng/dL)/(ng/mL/hr) | CCT  - ARR >20 (ng/dL)/(ng/mL/hr)  SIT  - post-infusion PAC >6 ng/dL  UFT  - PRA <2.0 ng/ml/h in post-loading blood sample |
| **Yang** | 2016 | ARR >24 (ng/dL)/(ng/mL/hr) with PAC>20 ng/dL | SIT  - post-infusion PAC >10 ng/dL |
| **Watanabe** | 2016 | N/A | N/A |
| **Hanslik** | 2015 | ARR >20-40 (ng/dL)/(ng/mL/hr)* | CCT  - PAC suppressed <30% after captopril  SIT  - post-infusion PAC >10 ng/dL  Fludrocortisone suppression test  - Upright PAC >6 ng/dL  Oral sodium loading  - 24h urine aldosterone excretion > 12 µg/24hr |
| **Turchi** | 2014 | (1) (a) Upright PAC above upper normal limits, and urinary aldosterone levels above upper normal limits; plus (b) a finding of low upright PRA (<1.0 ng/ml per h) plus (c) PAC after saline infusion higher or equal to 10 ng/dl; (2) (a) plus (c) plus normal upright PRA; (3) (b) plus (c) plus normal baseline upright values of serum aldosterone and urinary aldosterone; (4) (a) plus (b) plus suppressible serum aldosterone after saline infusion (i.e. < 10 ng/dl) plus evidence of an adrenal mass. | |
| **Savard** | 2013 | ARR >64 pmol/mIU (3.6 ng/ng) on 2 occasions and if PAC >550 pmol/L (20 ng/dL) in the standing or sitting position or >500 pmol/L (18 ng/dL) in the supine position  or urinary aldosterone excretion was >63 nmol/d (23 μg/d) | |
| **Fischer** | 2013 | ARR >50 ng/mU (sitting) | SIT  - ARR >50 ng/L |
| **Prejbisz** | 2013 | ARR >30 (ng/dL)/(ng/mL/hr) with PAC>15 ng/dL | CCT  - PAC suppressed <30% after captopril |
| **Somloova** | 2010 | ARR >30 (ng/dL)/(ng/mL/hr) with PRA <0.7 ng/mL/hr PAC>15 ng/dL | SIT  - post-infusion PAC >7 ng/dL |
| **Reinke** | 2010 | 3 of the following 6 criteria had to be present  1. Elevated ARR, or suppressed renin and elevated PAC in those patients without ARR  2. PAC >middle normal range 3. Abnormal confirmatory testing  4. Adrenal adenoma in histopathology  5. Blood pressure response to adrenalectomy  6. Blood pressure response to treatment with mineralocorticoid antagonist | |
| **Iacobellis** | 2010 | ARR >40 (ng/dL)/(ng/mL/hr) with suppressed PRA and PAC>15 ng/dL | SIT  - post-infusion PAC >5 ng/dL |
| **Ronconi** | 2009 | ARR >40 (ng/dL)/(ng/mL/hr) and PAC>15 ng/dL | SIT  - post-infusion PAC >7 ng/dL |
| **Fallo** | 2010 | ARR >40 (ng/dL)/(ng/mL/hr) with suppressed PRA and PAC>15 ng/dL | SIT  - post-infusion PAC >5 ng/dL |
| **Matrozova** | 2009 | ARR >64 pmol/mU (107 pmol/ng) on 2 occasions and the PAC >500 pmol/L  (18 ng/dL) in the supine position or 550 pmol/L (20 ng/dL) in the standing or sitting position or if urinary aldosterone excretion was >63 nmol/d (23 g/d) | |
| **Mosso** | 2007 | ARR >25 (ng/dL)/(ng/mL/hr) | UFT  - Upright PAC >5 ng/dL |
| **Fallo** | 2007 | ARR >40 (ng/dL)/(ng/mL/hr) with suppressed PRA and PAC>15 ng/dL | SIT  - post-infusion PAC >5 ng/dL |
| **Catena** | 2006 | ARR >20 (ng/dL)/(ng/mL/hr) with PAC>15 ng/dL | SIT  - post-infusion PAC >5 ng/dL |
| **Fallo** | 2006 | ARR >40 (ng/dL)/(ng/mL/hr) with suppressed PRA and PAC>15 ng/dL | SIT  - post-infusion PAC >5 ng/dL |
| **Widimsky** | 2001 | Decreased PRA, an increased PAC, increased ARR, postural, NaCl and dexamethasone tests and genetic screening for the exclusion of dexamethasone suppressible hyperaldosteronism (DSH)) as well as morphological methods (adrenal CT scan, adrenal venous sampling) | |

*Endocrine Society 2016 criteria

ARR: aldosterone-renin ratio, PAC: plasma aldosterone concentration, PRA: plasma renin activity, SIT: saline infusion test, CCT: captopril challenge test, UFT: upright furosemide loading test, N/A: data not available

**Table S3.1.** Risk of bias assessed by Joanna Briggs Institute (JBI) Critical Appraisal Tools for cross-sectional study

| **Question** | **Grewal 2021** | **Choudhary 2020** | **Manosroi 2020** | **Vujacik 2020** | **Zhang 2020** | **Monticone**  **2017** | **Murata**  **2017** | **Yang 2016** | **Watanabe 2016** | **Hanslik 2015** | **Savard 2013** | **Fischer 2013** | **Prejbisz 2013** |
| --- | --- | --- | --- | --- | --- | --- | --- | --- | --- | --- | --- | --- | --- |
| Were the criteria for inclusion in the sample clearly defined? | / | / | / | / | / | / | / | / | / | / | / | / | / |
| Were the study subjects and the setting described in detail? | / | / | / | / | / | / | / | / | / | / | / | / | / |
| Was the exposure measured in a valid and reliable way? | / | / | / | / | / | / | / | / | / | / | / | / | / |
| Were objective, standard criteria used for measurement of the condition? | / | / | / | / | / | / | / | / | / | / | / | / | / |
| Were confounding factors identified? | X | / | / | / | / | / | / | / | / | / | / | / | X |
| Were strategies to deal with confounding factors stated? | X | / | / | X | / | / | / | / | / | / | / | / | X |
| Were outcomes measured in a valid and reliable way? | / | N/A | N/A | N/A | N/A | N/A | N/A | N/A | N/A | / | N/A | / | / |
| Was appropriate statistical analysis used? | X | / | / | X | / | / | / | / | / | / | / | / | X |
| **Total** | **5** | **7** | **7** | **5** | **7** | **7** | **7** | **7** | **7** | **8** | **7** | **8** | **5** |
| **Risk of bias** | **Moderate** | **Low** | **Low** | **Moderate** | **Low** | **Low** | **Low** | **Low** | **Low** | **Low** | **Low** | **Low** | **Moderate** |

| **Question** | **Somloova 2010** | **Iacobellis 2010** | **Ronconi 2009** | **Fallo 2010** | **Matrozova 2009** | **Mosso 2007** | **Fallo 2007** | **Fallo 2006** | **Widimsky 2001** |
| --- | --- | --- | --- | --- | --- | --- | --- | --- | --- |
| Were the criteria for inclusion in the sample clearly defined? | / | / | / | / | / | / | / | / | / |
| Were the study subjects and the setting described in detail? | / | / | / | / | / | / | / | / | / |
| Was the exposure measured in a valid and reliable way? | / | / | / | / | / | / | / | / | / |
| Were objective, standard criteria used for measurement of the condition? | / | / | / | / | / | / | / | / | / |
| Were confounding factors identified? | / | / | / | / | / | / | / | / | / |
| Were strategies to deal with confounding factors stated? | / | / | / | / | / | / | / | / | / |
| Were the outcomes measured in a valid and reliable way? | / | / | / | N/A | / | / | / | / | / |
| Was appropriate statistical analysis used? | / | / | / | / | / | / | / | / | / |
| **Total** | **8** | **8** | **8** | **7** | **8** | **8** | **8** | **8** | **8** |
| **Risk of bias** | **Low** | **Low** | **Low** | **Low** | **Low** | **Low** | **Low** | **Low** | **Low** |

Low risk: scores ≥7

Moderate risk: scores 4-6

High risk: scores <4

**Table S3.2.** Risk of bias assessed by Joanna Briggs Institute (JBI) Critical Appraisal Tools for cohort study

| **Question** | **Catena**  **2006** | **Turchi**  **2014** | **Hundemer**  **2018** |
| --- | --- | --- | --- |
| Were the two groups similar and recruited from the same population? | / | / | / |
| Were the exposures measured similarly to assign people to both exposed and unexposed groups? | / | / | / |
| Was the exposure measured in a valid and reliable way? | / | / | / |
| Were confounding factors identified? | / | / | / |
| Were strategies to deal with confounding factors stated? | / | / | / |
| Were the groups/participants free of the outcome at the start of the study (or at the moment of exposure)? | N/A | N/A | / |
| Were the outcomes measured in a valid and reliable way? | N/A | / | / |
| Was the follow up time reported and sufficient to be long enough for outcomes to occur? | N/A | N/A | / |
| Was follow up complete, and if not, were the reasons to loss to follow up described and explored? | N/A | N/A | / |
| Were strategies to address incomplete follow up utilized? | N/A | N/A | / |
| Was appropriate statistical analysis used? | / | / | / |
| **Total** | **6** | **7** | **11** |
| **Risk of bias** | **Moderate** | **Moderate** | **Low** |

High risk: ≤49% of “yes” scores

Moderate risk: 50 to 69% of “yes” scores

Low risk: more than 70% of “yes” scores

**Table S3.3.** Risk of bias assessed by Joanna Briggs Institute (JBI) Critical Appraisal Tools for case-control study

| **Question** | **Reincke**  **2012** | **Huang**  **2021** |
| --- | --- | --- |
| Were the groups comparable other than the presence of disease in cases or the absence of disease in controls? | / | / |
| Were cases and controls matched appropriately? | / | / |
| Were the same criteria used for identification of cases and controls? | / | / |
| Was exposure measured in a standard, valid and reliable way? | / | / |
| Was exposure measured in the same way for cases and controls? | / | / |
| Were confounding factors identified? | / | / |
| Were strategies to deal with confounding factors stated? | / | / |
| Were outcomes assessed in a standard, valid and reliable way for cases and controls? | N/A | N/A |
| Was the exposure period of interest long enough to be meaningful? | N/A | N/A |
| Was appropriate statistical analysis used? | / | / |
| **Total** | **8** | **8** |
| **Risk of bias** | **Low** | **Low** |

High risk: ≤49% of “yes” scores

Moderate risk: 50 to 69% of “yes” scores

Low risk: more than 70% of “yes” scores

**Table S4.** Egger’s regression analysis

| **Outcomes** | **p-value** |
| --- | --- |
| All glycemic abnormalities | 0.122 |
| Diabetes mellitus | 0.496 |
| Impaired fasting glucose | 0.360 |
| Impaired glucose tolerance | 0.140 |
| Fasting blood glucose | 0.078 |
| HbA1c | 0.760 |
| 2-hr oral glucose tolerance test | 0.151 |
| HOMA-IR | 0.126 |
| HOMA- ß | 0.538 |
| AUC of glucose | 0.058 |
| AUC of insulin | 0.252 |
| QUICKI | 0.199 |

**Table S5.** Grading of Recommendations Assessment, Development and Evaluation (GRADE) rating

| **Outcome** | **Certainty assessment** | | | | | | | **Absolute effect (95% CI)** | **Certainty** |
| --- | --- | --- | --- | --- | --- | --- | --- | --- | --- |
|  | **No. of studies** | **Study design** | **Risk of bias** | **Inconsistency** | **Indirectness** | **Imprecision** | **Other considerations** |  |  |
| All glycemic abnormalities | 18 | Observational | Not serious | Serious | Serious | Not serious | None | RR 1.54 (1.20,1.98) | Very low |
| DM | 15 | Observational | Not serious | Not serious | Serious | Not serious | None | RR 1.27 (1.08,1.49) | Very low |
| IGT | 5 | Observational | Not serious | Not serious | Serious | Not serious | None | RR 2.99 (1.74,5.16) | Very low |
| IFG | 4 | Observational | Not serious | Serious | Serious | Serious | None | RR 1.70 (0.55, 5.26) | Very low |
| FBG | 10 | Observational | Not serious | Not serious | Serious | Serious | None | SMD 0.07 (-0.05, 0.20) | Very low |
| HbA1c | 6 | Observational | Not serious | Serious | Serious | Serious | None | SMD 0.01 (-0.23, 0.25) | Very low |
| 2-hr OGTT | 5 | Observational | Not serious | Not serious | Serious | Serious | None | SMD -0.05 (-0.35, 0.25) | Very low |
| HOMA-IR | 10 | Observational | Not serious | Serious | Serious | Serious | None | SMD 0.18 (-0.28, 0.64) | Very low |
| HOMA-ß | 5 | Observational | Not serious | Not serious | Serious | Serious | None | SMD -0.44 (-0.62, -0.26) | Very low |
| AUC glucose | 3 | Observational | Not serious | Serious | Serious | Serious | None | SMD 0.83 (-0.23, 1.90) | Very low |
| AUC insulin | 4 | Observational | Not serious | Serious | Serious | Serious | None | SMD 0.43 (-0.54, 1.41) | Very low |
| QUICKI | 3 | Observational | Not serious | Serious | Serious | Serious | None | SMD -0.90 (-2.62, 0.81) | Very low |

**Figure S1.** Forest plots of subgroup analyses of all glycemic abnormalities comparing primary aldosteronism and essential hypertension patients: ethnicity (A) and demographic data matching (B).


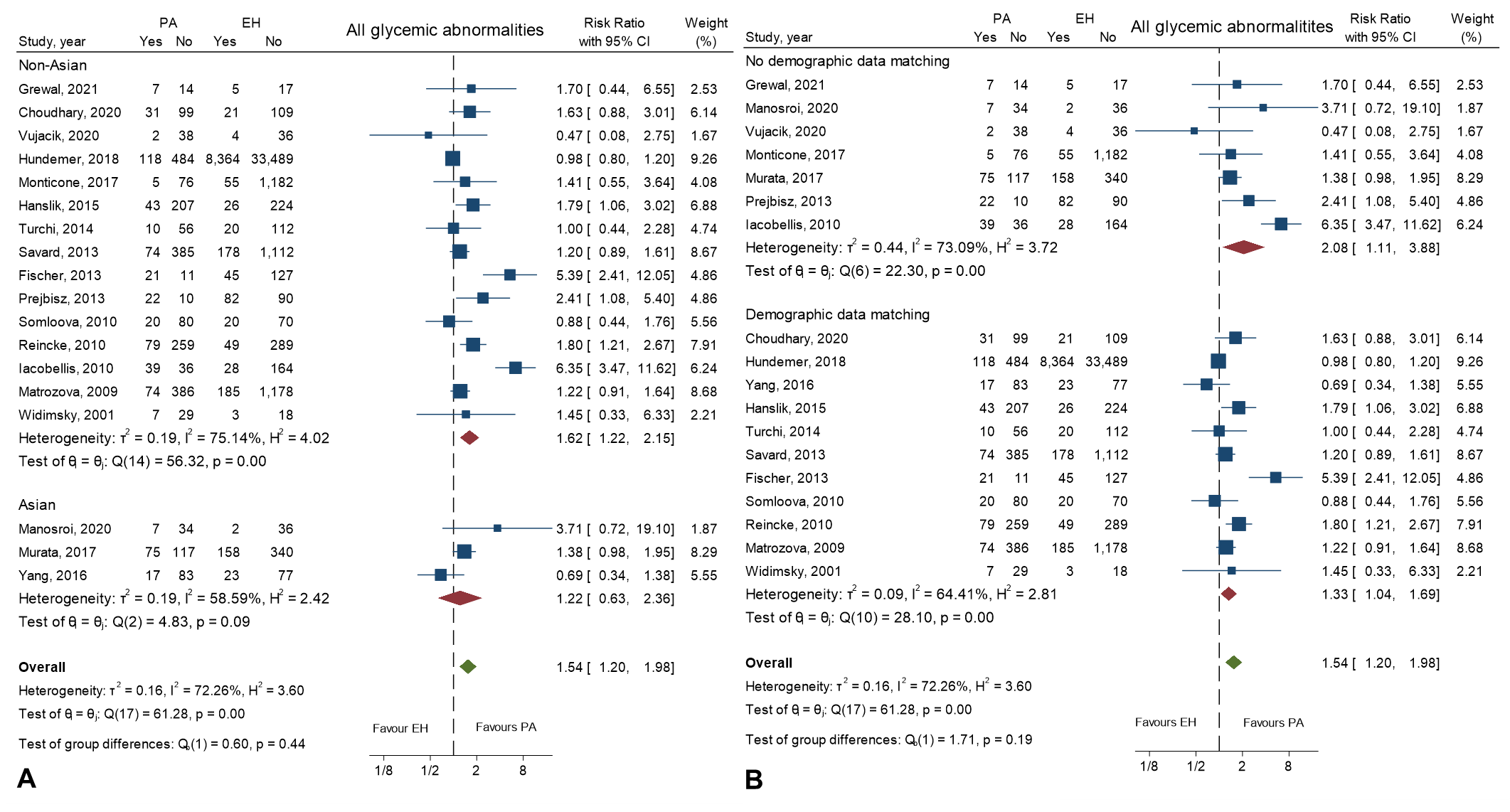


**Figure S2.** Funnel plots of studies with the outcomes of glycemic abnormalities: all glycemic abnormalities (A), diabetes mellitus (B), impaired glucose tolerance (C) and impaired fasting glucose (D)


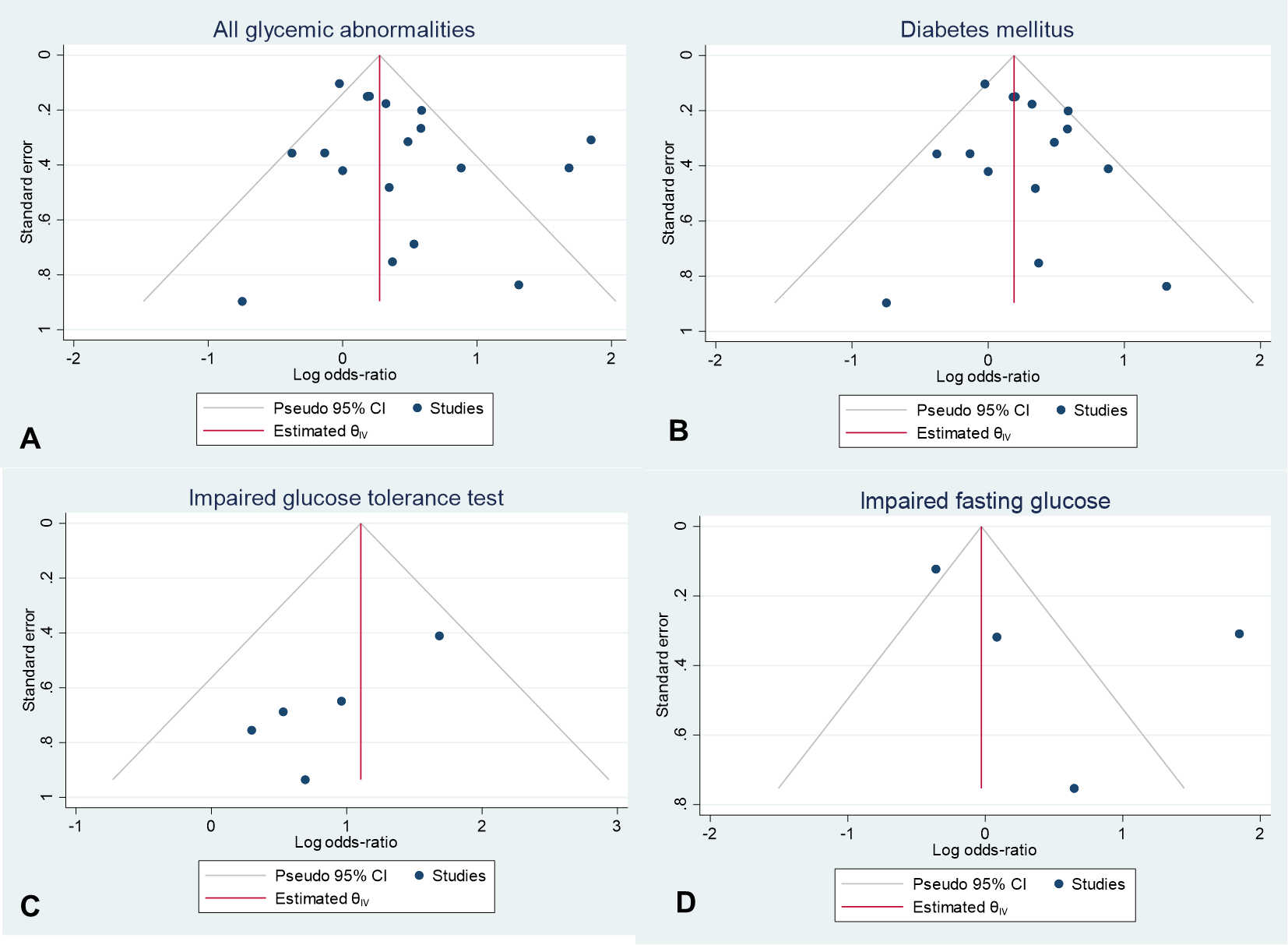


**Figure S3.** Funnel plots of the studies with the outcomes of glucose metabolic profiles: fasting blood glucose (A), HbA1c (B), 2-hr oral glucose tolerance test (2-hr OGTT) (C), homeostatic model assessment of insulin resistance (HOMA-IR) (D), homeostatic model assessment of ß-cell function (HOMA-ß) (E), area under the curve (AUC) of glucose (F), AUC of insulin (G) and the quantitative insulin-sensitivity check index (QUICKI) (H).


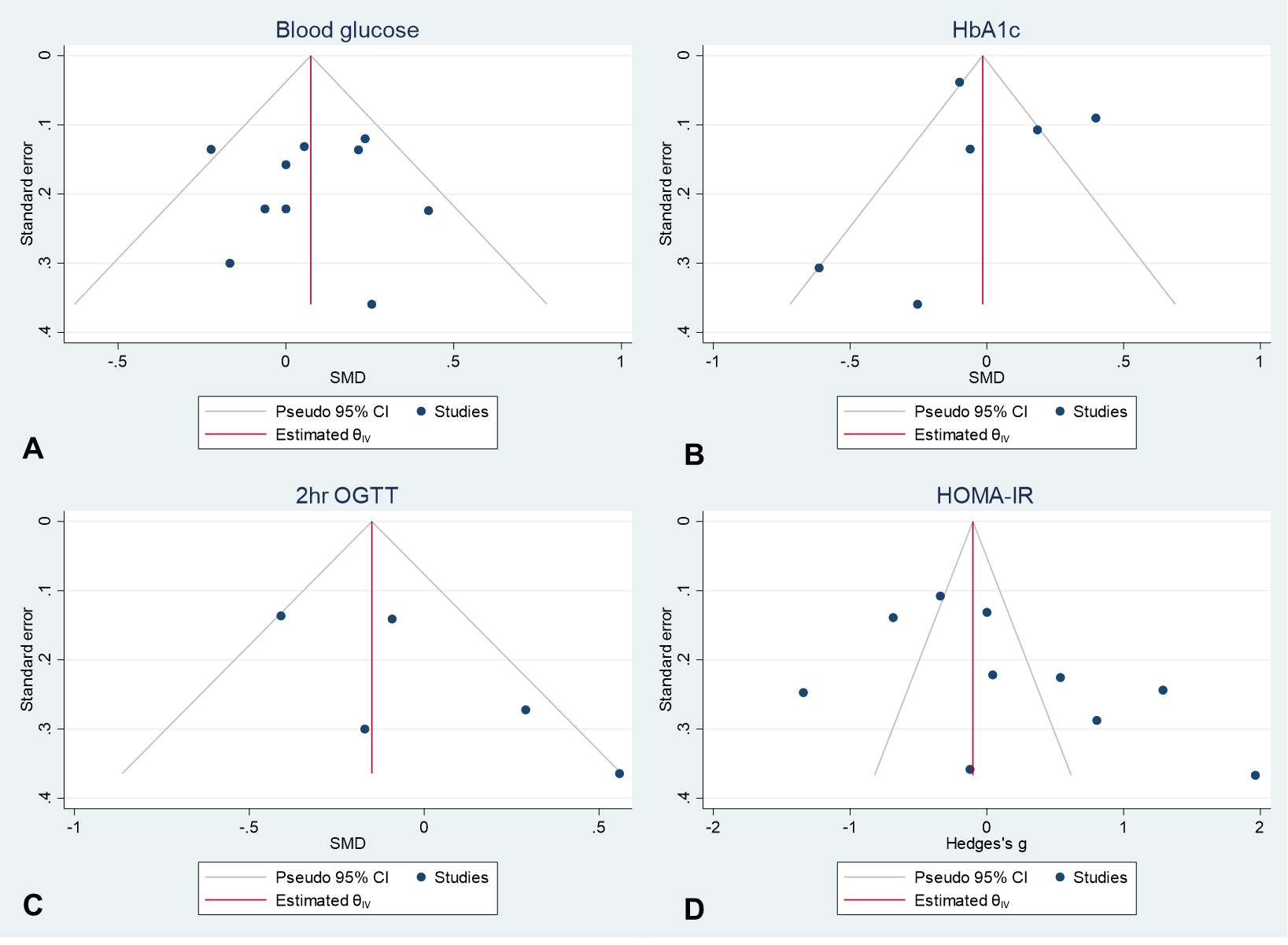


**Figure S3. (continued)** Funnel plots of the studies with the outcomes of glucose metabolic profiles: fasting blood glucose (A), HbA1c (B), 2-hr oral glucose tolerance test (2-hr OGTT) (C), homeostatic model assessment of insulin resistance (HOMA-IR) (D), homeostatic model assessment of ß-cell function (HOMA-ß) (E), area under the curve (AUC) of glucose (F), AUC of insulin (G) and the quantitative insulin-sensitivity check index (QUICKI) (H).


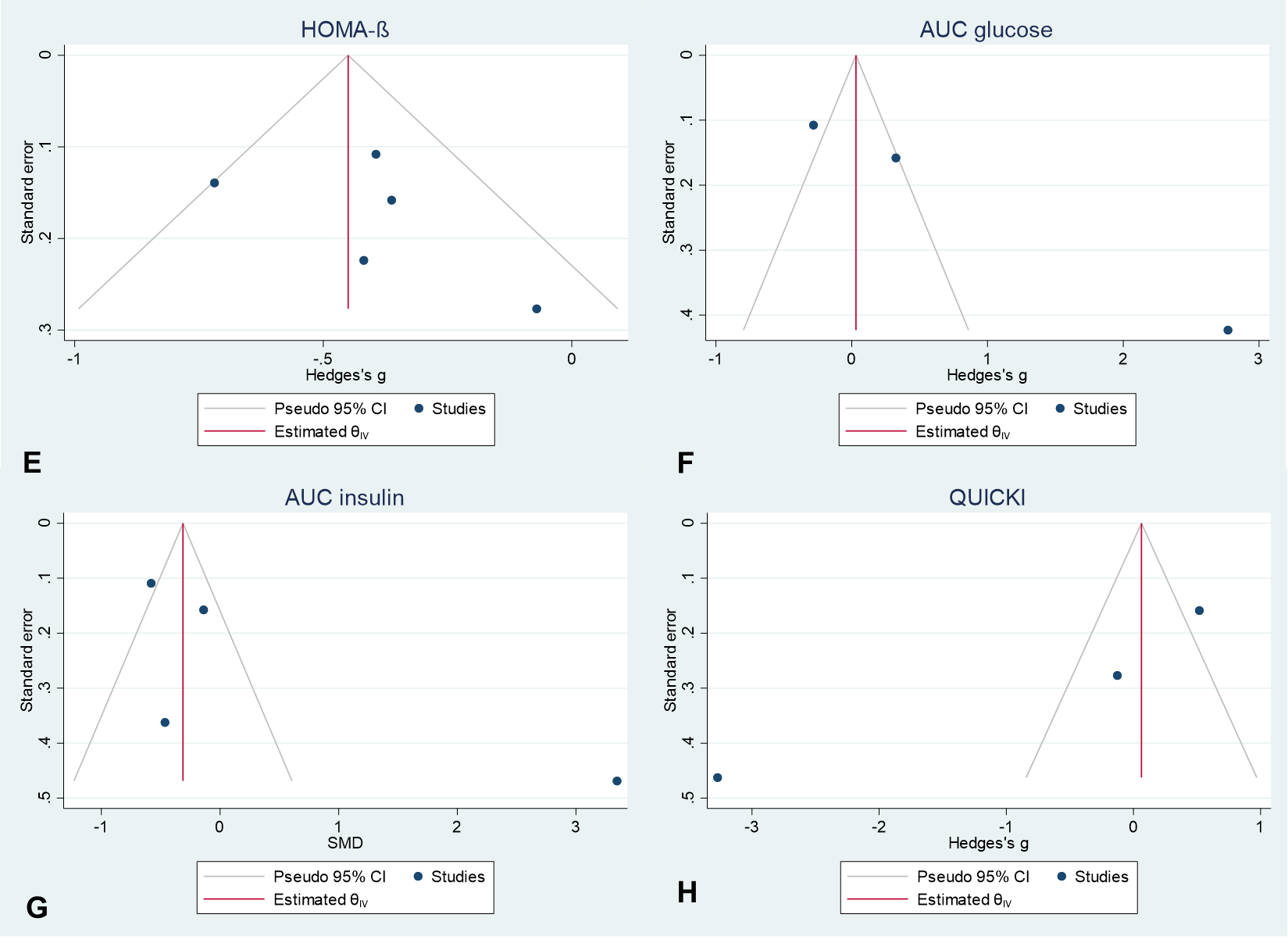

Supplement: Supplementary file 1 [file DataSheet_1.docx]
